# Pharmacy students’ use of e-learning resources: a repeated cross-sectional study in 2021 and 2026

**DOI:** 10.1186/s13104-026-07878-4

**Published:** 2026-05-20

**Authors:** Seeba Zachariah, Alaa Fouda, Zahraa Altamimi, Asma Qayyum, Fatimaalzhra Hayder, Juny Sebastian

**Affiliations:** Pharmacy Practice, College of Pharmacy, Gulf Medical University, Ajman, United Arab Emirates

**Keywords:** E-resources, Digital learning, E-learning, Student preferences, Pharmacy education, Digital library

## Abstract

**Objective:**

Understanding pharmacy students’ engagement with e‑learning resources is essential for supporting evidence‑based learning. The COVID‑19 pandemic temporarily intensified reliance on digital materials, creating a unique opportunity to examine how usage patterns evolve once mandatory digital access ends. This study compared pharmacy students’ use and preferences for e‑learning resources in 2021, when digital access was required, and in 2026, when usage was voluntary within a more mature digital ecosystem.

**Results:**

A repeated cross‑sectional survey was administered to all students enrolled in a five‑year entry‑to‑practice pharmacy program at a medical university in the United Arab Emirates. Data were collected in November–December 2021 and January–February 2026. A total of 171 students participated in 2021 and 134 in 2026. While core reading attitudes remained stable, digital engagement increased significantly over time (*p* = 0.01). Students in 2026 reported more frequent e‑library use, greater electronic reading time, and substantially higher utilization of AccessPharmacy (22.2% to 67.2%; *p* < 0.001), ClinicalKey (25.1% to 42.5%; *p* = 0.001), and UpToDate (36.3% to 81.3%; *p* < 0.001). Perceptions of accessibility and learning effectiveness also shifted toward e‑books. These findings indicate sustained and growing reliance on digital learning resources well beyond the pandemic period.

**Supplementary Information:**

The online version contains supplementary material available at 10.1186/s13104-026-07878-4.

## Introduction

The COVID-19 pandemic accelerated a global shift toward digital learning and the widespread adoption of academic e-resources across higher education institutions, including medical and health sciences universities. Academic libraries rapidly expanded access to e-books, online journals, drug information databases, and clinical guidelines to support remote learning and maintain educational continuity during lockdowns and campus closures. Studies across health professions reported substantial increases in students’ reliance on digital platforms, online course materials, and virtual library services during the pandemic, reflecting a broader transformation in information-seeking behaviors and resource utilization patterns [1–5]. Pharmacy education, in particular, experienced a marked transition to technology-enhanced learning, with students reporting increased engagement with digital content and online instructional modalities [6–8].

Research conducted during the pandemic highlighted both the opportunities and challenges associated with this rapid digital transition. Systematic reviews and cross-sectional studies documented pharmacy students’ perceptions of e-learning strategies, noting improvements in flexibility and accessibility but also identifying barriers such as digital fatigue, reduced interaction, and variable digital literacy [1, 9–12]. Academic libraries similarly reported shifts in usage patterns, with increased demand for electronic collections and decreased reliance on physical materials, trends consistent with global observations in higher education [13–15]. These changes were influenced not only by institutional mandates but also by students’ evolving preferences for convenience, immediate access, and the integration of digital tools into their study routines [16–18].

By 2026, the educational landscape had further evolved. Artificial intelligence (AI) enabled tools had emerged in some academic e-resources. The extent of AI use among pharmacy students remains underexplored. Recent literature suggests that AI-supported learning tools are increasingly shaping students’ study habits, information retrieval strategies, and expectations for digital resource functionality [19–20]. This shift marks a move from pandemic-driven digital adoption to a more technologically mature environment. Now, AI and advanced digital platforms coexist with traditional academic resources.

Given these developments, it is essential to understand whether the use of e-learning resources has changed between 2021 and 2026. The present study aims to compare the use and preferences of e-resources and hard-copy materials among entry-to-practice pharmacy students at a medical university in the United Arab Emirates (UAE), using repeated surveys conducted in 2021 and 2026.

## Methods

### Study design and setting

A repeated cross-sectional survey was conducted at a medical university in the UAE to assess trends in the use and preferences of e-learning resources among entry-to-practice pharmacy students. Data were collected at two distinct time frames: November–December 2021, representing the late COVID-19 period when digital resources were institutionally emphasized from 2020; and January–February 2026, when AI tools began to appear on some academic platforms. The study included electronic resources provided by the university library, such as e-books, drug databases, e-journals, and clinical guidelines and hard-copy materials available on campus. E-resources were accessed via the university’s e-library using individual logins.

### Study population

The study targeted all undergraduate pharmacy students enrolled across the five years of the program at both time points. As this was a population-based survey, all eligible students were invited to participate. No sample size calculation was performed. Inclusion criteria were enrollment in the pharmacy program during each survey period and willingness to complete the questionnaire. Participation was voluntary and anonymous.

### Survey instrument

A structured, self-administered questionnaire was used in both 2021 and 2026. The survey was developed specifically for this study; its content validity was reviewed by two subject-matter experts and piloted with a small group of students to ensure clarity and feasibility. The instrument included items on frequency of e-learning resource use, preferences for electronic or hard-copy materials, perceived usefulness of resource types, and factors influencing selection. The full questionnaire is available in Supplementary File 1. The 2026 survey retained the core items from 2021 for direct comparison, but did not include detailed questions on AI use, as the focus remained on e-learning resources. Minor wording adjustments were made for clarity without altering the measured constructs.

### Data collection procedures

The survey was distributed electronically through the university’s learning management system. Students received an invitation link and two reminders during each data collection period. No personal identifiers were collected. All responses were automatically captured in a secure institutional database accessible only by the research team.

### Data analysis

Data were analyzed using IBM SPSS Statistics version 30. Descriptive statistics summarized demographic data and resource use patterns. Categorical variables from 2021 to 2026 were compared using chi-square or Mann–Whitney U tests to assess differences in e-resource use and preferences over time. Statistical significance was set at *p* < 0.05.

### Ethical considerations

Ethical approval was obtained from the Gulf Medical University Institutional Review Board before data collection (IRB/COP/STD/75/Oct-2021). All study procedures were conducted in accordance with relevant guidelines and regulations, including the principles of the Declaration of Helsinki. Participation was voluntary, and electronic informed consent was obtained from all participants. No identifiable information was collected. Confidentiality and anonymity were guaranteed.

## Results

The survey was filled out by 171 students in 2021 and 134 students in 2026, representing 80.3% and 70.5% of the pharmacy student population, respectively. The graduating students of 2026 also completed the survey in 2021 when they were in their first year; all other students who completed the surveys in 2021 and 2026 belonged to different cohorts. While in the 2021 survey, the percentage of respondents was higher in years 1 and 2, in 2026, the response rate was higher in year 5, with years 3 and 4 showing similar response rates (Table 1).

**Table 1:** Survey response rate across 5 years of the pharmacy program

Students	Number and Percentage of Response			
	2021	2021%	2026	2026%
Year of Study				
1	45	26.3%	24	17.9%
2	41	24.0%	17	12.7%
3	40	23.4%	34	25.4%
4	31	18.1%	23	17.2%
5	14	8.2%	36	26.9%
Total	171	100	134	100

A Mann–Whitney U test showed a statistically significant difference in age between the 2021 and 2026 cohorts (*p* < 0.001). Students in 2026 were generally older (Median = 21, Interquartile range, IQR = 20–22) than those in 2021 (Median = 20, IQR = 18–21). Gender distribution did not differ significantly between cohorts, as assessed by a Chi-Square test (*p* = 0.46). Overall, the combined female population in both surveys was 77.4%, while males accounted for 22.6%.

As shown in Table 2, several variables remained consistent across the two survey years.

Students’ general enjoyment of reading, enjoyment of reading for pleasure, and preference for reading for academic purposes showed no meaningful differences. Behaviors related to printing eBooks, annotating PDFs, and borrowing textbooks from the library were also stable. Additionally, the proportion of students who used the university e-library at least occasionally was similar across years, as were perceptions of which format (eBooks or printed textbooks) was more expensive. These findings suggest that students’ core reading attitudes and several routine reading behaviors remained stable over time.

**Table 2:** Similar reading habits and perceptions among 2021 and 2026 survey respondents

Item	Response	Total (*n* = 305)		2021(*n* = 171)			2026 (*n* = 134)		Chi-square p-value
		Total	Percent (%)	Count	Percent (%)		Count	Percent (%)	
Enjoy Reading (General)	No	86	28.2	43	25.1		43	32.1	0.18
	Yes	219	71.8	128	74.9		91	67.9	
Enjoy Reading for Pleasure	No	83	27.2	45	26.3		38	28.4	0.69
	Yes	222	72.8	126	73.7		96	71.6	
Enjoy Reading for Academic purposes	No	52	17.0	28	16.4		24	17.9	0.72
	Yes	253	83.0	143	83.6		110	82.1	
Often Print eBooks	No	186	61.0	112	65.5		74	55.2	0.07
	Yes	119	39.0	59	34.5		60	44.8	
Annotate PDF (Highlight/Notes)	No	47	15.4	31	18.1		16	11.9	0.13
	Yes	258	84.6	140	81.9		118	88.1	
Use University e-Library	No	55	18.0	36	21.1		19	14.2	0.12
	Yes	250	82.0	135	78.9		115	85.8	
Borrow textbooks from Library	No	188	61.6	106	62.0		82	61.2	0.89
	Yes	117	38.4	65	38.0		52	38.8	
Which one is expensive	eBooks	48	15.7	22	12.9		26	19.4	0.12
	Textbooks	257	84.3	149	87.1		108	80.6	

There were multiple preferences, and usage varied significantly among students who completed the 2021 and 2026 surveys. The overall multiple‑response chi‑square test for top resource use showed a significant difference between 2021 and 2026 (*p* = 0.01), indicating that students’ preferred learning platforms changed over time. More information is provided in Table 3.

**Table 3:** Differing preferences and usages among 2021 and 2026 survey respondents

Item	Response	Total (*n* = 305)		2021(*n* = 171)		2026 (*n* = 134)		Chi-square p-value
		Total	Percent (%)	Count	Percent (%)	Count	Percent (%)	
Preferred Format	eBook	179	58.7	115	67.3	64	47.8	< 0.001
	Hard copy	126	41.3	56	32.7	70	52.2	
Format Often Used	eBook	252	82.6	163	95.3	89	66.4	< 0.001
	Hard copy	53	17.4	8	4.7	45	33.6	
Frequency of using e-Library	Never	31	10.2	20	11.7	11	8.2	< 0.001
	Once per semester	43	14.1	35	20.5	8	6.0	
	Once in 3 months	48	15.7	33	19.3	15	11.2	
	Monthly	78	25.6	56	32.7	22	16.4	
	Weekly	77	25.2	19	11.1	58	43.3	
	Daily	28	9.2	8	4.7	20	14.9	
Weekly Hours Using Electronic Devices	Less than 10 h	216	70.8	137	80.1	79	59.0	< 0.001
	10 to 20 h	72	23.6	31	18.1	41	30.6	
	More than 20 h	17	5.6	3	1.8	14	10.4	
Accessibility of eBooks vs. Print	Both easily accessible	98	32.1	59	34.5	39	29.1	0.04
	eBooks more accessible	150	49.2	74	43.3	76	56.7	
	Textbooks more accessible	46	15.1	33	19.3	13	9.7	
	Both difficult	11	3.6	5	2.9	6	4.5	
Format Best for Learning.	Equal	108	35.4	53	31.0	55	41.0	< 0.001
	eBooks are more effective	72	23.6	27	15.8	45	33.6	
	Printed texts are more effective	125	41.0	91	53.2	34	25.1	

Patterns of e-library engagement also changed. Although overall use remained high, the frequency of e-library use shifted markedly, with the 2026 cohort reporting substantially higher weekly and daily use. Students in 2026 also spent significantly more hours per week using electronic devices for reading, with increases in both the 10–20-hour and > 20-hour categories. AccessPharmacy use increased dramatically from 22.2% in 2021 to 67.2% in 2026 (*p* < 0.001), representing the most substantial shift among all resources. ClinicalKey use also rose significantly (25.1% to 42.5%; *p* = 0.001). UpToDate showed a similar pattern, with usage increasing from 36.3% in 2021 to 81.3% in 2026 (*p* < 0.001) (Table 4). These findings suggest a strong movement toward institutional digital databases and clinical decision-support tools.

**Table 4:** Differing preference for electronic devices or database between 2021 and 2026 respondents

Item	Devise/Database	Total		2021		2026		Chi-square p value	Multiple response Chi-square p value
		Count	%	Count	%	Count	%		
Electronic Device Use	Smartphone	127	41.6	79	46.2	48	35.8	0.07	< 0.001
	Laptop/Desktop	214	70.2	124	72.5	90	67.2	0.31	
	Tablet	107	35.1	42	24.6	65	48.5	< 0.001	
	E-book Reader	14	4.6	6	3.5	8	6.0	0.31	
	Only Print	28	9.2	21	12.3	7	5.2	0.03	
Use of the Database	AccessPharmacy	128	42.0	38	22.2	90	67.2	< 0.001	0.01
	ClinicalKey	100	32.8	43	25.1	57	42.5	0.001	
	UpToDate	171	56.1	62	36.3	109	81.3	< 0.001	

Perceptions of accessibility and learning effectiveness also evolved. Students in 2026 were more likely to report that eBooks were easier to access and less likely to view printed textbooks as more accessible. Similarly, beliefs about the format best suited for learning shifted: fewer students favored printed texts alone, and more viewed eBooks or a combination of formats as equally or more effective.

## Discussion

The present study examined changes in pharmacy students’ use and preferences for e-learning resources between 2021 and 2026, revealing a marked increase in e-resource usage, time spent engaging with digital materials, and stronger preferences for e-books in the later survey period. The observed increase in the use of advanced clinical databases in 2026 may partly reflect differences in cohort composition, as a higher proportion of senior students, who are more likely to engage with such resources, were represented in the later sample. This suggests that the findings should be interpreted with caution, as they may not solely indicate a temporal change but also differences in the distribution of year of study. These findings align with global reports documenting growth in digital resource utilization in higher education following the COVID-19 pandemic, particularly within health professions programs [21–23].

Although students at the institution were already familiar with e-resources prior to the pandemic, the mandatory shift to digital platforms in 2020 partially continued to 2021, implemented to reduce infection risks associated with shared hard-copy materials, appears to have accelerated long-term adoption patterns. Similar trends have been observed internationally, where pandemic-driven digital transitions catalyzed enduring changes in students’ information-seeking behaviors and academic resource preferences [24–26].

While overall engagement with digital resources remained high, the pattern of use in 2026 reflects a reconfiguration of preferences rather than uniform growth, with some formats (e.g., eBooks) showing a notable decline alongside increases in other resource types. The shift toward e-books and online databases observed in 2026 is consistent with studies showing that pharmacy and medical students increasingly value the accessibility, searchability, and portability of digital materials [27–29]. While many students initially preferred printed books for their tactile qualities and perceived benefits for concentration and retention, evidence suggests that familiarity with digital platforms and improvements in interface design have contributed to greater acceptance of e-learning resources over time [30–33]. The increased time spent on the e-library in 2026 may also reflect enhanced digital literacy and greater integration of electronic resources into coursework, as reported in other health sciences programs [34–37].

Although the 2026 survey did not explicitly assess artificial intelligence (AI) use, the broader educational landscape has been shaped by the rapid emergence of AI-enabled tools, which may have indirectly influenced students’ confidence in digital resources. Literature indicates that AI-supported learning systems can enhance information retrieval, personalize study strategies, and improve students’ perceptions of the usefulness of digital platforms [38–39]. At the same time, concerns about the accuracy and reliability of freely available AI tools have been documented, leading some learners to place greater trust in institutionally curated e-resources, which are perceived as more authoritative and evidence-based [40–42]. This dynamic may partly explain the stronger preference for library-provided digital materials observed in 2026.

The shift toward e-resources also reflects broader transformations in academic libraries, which have increasingly emphasized digital collections, remote access, and user-centered online services in the post-pandemic era [43–46]. Students expect seamless access to electronic materials and often prioritize digital formats even when print options remain available [47–48]. The findings of this study reinforce these trends, suggesting that the pandemic served as a catalyst for long-term changes in resource use behaviors among pharmacy students. While the COVID-19 pandemic accelerated digital learning in academic settings, a parallel transformation occurred in healthcare environments, where hospitals rapidly adopted e-learning platforms and digital resources to support continuous professional training, clinical updates, and real-time communication.

Overall, the results demonstrate a clear progression from pandemic-driven necessity to post-pandemic preference, with pharmacy students in 2026 showing stronger engagement with and reliance on e-resources. Continued monitoring of these trends, including the evolving role of AI-enhanced tools, will be important for informing library services, curriculum design, and digital resource investment strategies in pharmacy education.

## Limitations

A few limitations should be acknowledged when interpreting these findings. The study relied on self-reported survey data, which may be subject to recall bias or social desirability bias, particularly regarding time spent on the e-resources or perceived effectiveness of different resource formats. Because the design was cross-sectional at two time points rather than longitudinal, changes observed between 2021 and 2026 cannot be attributed to a trend over the years. Additionally, the higher e-resource use observed in 2026 may reflect cohort effects (such as differences in baseline digital literacy or curricular changes) rather than pandemic-induced behavior change. The survey also did not capture detailed information about students’ use of emerging AI-enabled tools, which became more prominent by 2026 and may have influenced attitudes toward digital resources in ways not directly measured. Some participants did not use the institutional library, but their responses are still included because they use similar other resources. The response rate in 2026 was slightly less than in 2021, but still sufficient for analysis. Finally, the study was conducted at a single institution, which may limit generalizability to other pharmacy programs with different digital infrastructures, curricular emphases, or library systems.

## Conclusion

The findings indicate increased use and preference for e-resources in 2026 compared to 2021. However, the difference may partly reflect cohort effects, including variations in digital literacy or curricular exposure. Students not only increased their use of the e-learning resources and time spent reading on electronic devices, but also demonstrated a strong shift toward platforms such as AccessPharmacy, ClinicalKey, and UpToDate. These changes mirror global trends in health professions education. Improved digital literacy, better platform design, and greater integration of electronic materials into coursework have contributed to long-term adoption of digital learning tools. The growing trust in institutionally curated resources may reflect broader concerns about the reliability of freely available AI tools. This reinforces the value of authoritative, evidence-based digital collections.

These results suggest pharmacy programs and libraries must strengthen digital infrastructure, ensure seamless access to e-resources, and expand digital information training. Libraries should improve user-centered online services, integrate digital resource instruction, and monitor emerging tools like AI to meet evolving student needs. Ongoing evaluation of digital engagement will guide investment and support evidence-based learning.

## Electronic Supplementary Material

Below is the link to the electronic supplementary material.

Supplementary Material 1.

## Data Availability

Data is available from corresponding author upon request.

## Abbreviations

AI: Artificial intelligence
COVID‑19: Coronavirus disease 2019
IQR: Interquartile range
UAE: United Arab Emirates
