# Supplementary material for: Pharmacy students’ use of e-learning resources: a repeated cross-sectional study in 2021 and 2026
Source: BMC Res Notes. 2026 May 20;19:296. doi: 10.1186/s13104-026-07878-4 (PMC13366804; doi:10.1186/s13104-026-07878-4)
Supplement: Supplementary file 1 — Supplementary Material 1. [file 13104_2026_7878_MOESM1_ESM.pdf]

E-book/Textbook use preference among Pharmacy students

* Indicates required question

1. Email *

2. **Informed Consent** *

This study aims to understand Pharmacy students' e-book use preferences. Your email ID will be collected only to prevent duplicate submissions and calculate response rates; it will not be linked to your survey answers. All responses will remain confidential, reported in aggregate, and used solely for research purposes. Participation in this 5-minute survey is voluntary, and you may withdraw at any time. Please use your official GMU email.

Thank you!

Proceeding indicates your informed consent to participate.

Mark only one oval.

- Yes, I agree to participate. *Skip to question 3*
- No, I do not agree. *Skip to section 2 (Exit)*

Exit

Survey

3. Gender: *

Mark only one oval.

- Female
- Male

4. Age in years *

5. Pharmacy program *

Mark only one oval.

- BPharm/PharmD
- MCP
- PGPharmD
- MDD
- Diploma in Pharmacy

6. Semester: *

Mark only one oval.

- 1
- 2
- 3
- 4
- 5
- 6
- 7
- 8
- 9
- Clinical Rotations

7. Generally, do you like (enjoy) reading? *

Mark only one oval.

- Yes
- No

8. Do you like reading for for nonacademic purposes? *

Mark only one oval.

- Yes
- No

9. Average time spent reading for Non-academic purposes (hours per week) *

Mark only one oval.

- Less than 10 hours
- 10-20 hours
- More than 20 hours

10. Do you spent time for reading for academic purposes? *

Mark only one oval.

- Yes
- No

11. Average time spent reading for academic purposes (hours per week) *

Mark only one oval.

- Less than 10 hours
- 10 to 20 hours
- More than 20 hours

12. Generally, which format do you prefer? *

Mark only one oval.

- Hard Copy (Printed books)
- e-book/ digital format (including online databases and digital libraries)

13. Which format do you use more often? *

Mark only one oval.

- Hard copy
- e-book

14. Do you often **print** pages or sections from e-books for your study purposes? *

Mark only one oval.

- Yes
- No

15. Do you usually **save PDF** documents so you can underline, highlight, or add notes while studying? *

Mark only one oval.

- Yes
- No

16. Have you used GMU e-library and online databases ? *

Mark only one oval.

- Yes
- No

17. How often do you use GMU e-library? *

Mark only one oval.

- Daily
- Weekly
- Monthly
- Once in 3 months
- Once per semester
- Never

18. Have you ever borrowed physical textbooks from the university library? *

Mark only one oval.

- Yes
- No

19. What type of electronic device do you usually use for reading e-books? *

Check all that apply.

- [ ] Smartphone
- [ ] PC/Laptop
- [ ] Tablet
- [ ] e-book reader(eg. Kindle)
- [ ] None — I prefer printed materials

20. How many **hours per week** do you use electronic devices for reading e-books or databases? (Smartphone, PC/laptop, tablet/iPad, or e-book reader such as Kindle) *

Mark only one oval.

- Less than 10 hours
- 10 -20 hours
- More than 20 hours

21. How would you rate the **accessibility** of e-books and printed textbooks for your studies? Please select the option that best matches your experience. *

Mark only one oval.

- E-books are more accessible
- Printed textbooks are more accessible
- Both e-books and printed textbooks are easily accessible
- Both e-books and textbooks are difficult to access.

22. In your opinion, which format is more expensive for your studies? *

Mark only one oval.

- E-books
- Textbooks

23. Which format best supports your learning (remembering, integrating information, and recalling content during exams)? * Please select the option that best matches your opinion.

Mark only one oval.

- E-books and printed textbooks are equal
- E-books are more effective than printed textbooks
- Printed textbooks are more effective than e-books
- E-books are not effective for my learning

24. For the textbook **DiPiro's Pharmacotherapy: A Pathophysiologic Approach**, which format(s) do you use for studying? *

Select all that apply.

Check all that apply.

- [ ] I own the printed (hardcopy) textbook
- [ ] I borrowed the printed textbook from the library
- [ ] I have a PDF copy of this textbook
- [ ] I access the textbook through the university e-library
- [ ] I print selected chapters or pages when needed
- [ ] I do not use this textbook in any format

25. Which are your three favorite databases in the GMU e-library? *

Check all that apply.

- [ ] AccessPharmacy
- [ ] ClinicalKey
- [ ] Medicines Complete (BNF)
- [ ] UpToDate
- [ ] TRIP database
- [ ] MEDLINE complete
- [ ] Lecturio Medical

26. What do you like and dislike about e-books for your studies? *

27. What do you like and dislike about using printed (hardcopy) textbooks? *

This content is neither created nor endorsed by Google.

Google Forms
